# A bi-variate framework to model microbiome resilience in healthy dogs

**DOI:** 10.3389/fvets.2025.1486679

**Published:** 2025-04-02

**Authors:** Fabio Mainardi, Marc Garcia-Garcera, Andrea K. Nash

**Affiliations:** ^1^Nestlé Institute of Health Sciences, Nestlé Research, Lausanne, Switzerland; ^2^Department of Gastrointestinal Health, Nestlé Institute of Health Sciences, Nestlé Research, Lausanne, Switzerland; ^3^Nestlé Research, St. Louis, MO, United States

**Keywords:** functional principal component analysis, canine microbiome, microbiome stability, microbiome recovery, exercise stress

## Abstract

**Introduction:**

Ecological resilience is the capacity of an ecosystem to maintain its state and recover from disturbances. This concept can be applied to the gut microbiome as a marker of health.

**Methods:**

Several metrics have been proposed to quantify microbiome resilience, based on the prior choice of some salient feature of the trajectories of microbiome change. We propose a data-driven approach based on compositional and functional data analysis to quantify microbiome resilience. We demonstrate the validity of our approach through applications to sled dogs undergoing three types of exercise: running on an exercise wheel, pulling an all-terrain vehicle, and pulling a sled.

**Results:**

Microbiota composition was clearly impacted by each exercise type. Log-ratio analysis was utilized for dimensionality reduction and identified 33 variables (taxa) explaining 90% of the variance. Functional principal component analysis identified two scores (FPCA 1 and FPCA2) which explained 76% and 19% of the variability of the trajectories, respectively. More resilient trajectories corresponded to low values of FPCA1 and FPCA2 values close to zero. Levels of chemokines MCP-1 and KC-like, which increased significantly after exercise and returned to pre-exercise levels within 24 h, were significantly associated with FPCA scores as well.

**Discussion:**

To our knowledge, this is the first study proposing a principled approach to quantify microbiome resilience in healthy dogs and associate it with immune response to exercise-related stress.

## Introduction

The gut microbiota is a diverse community of microbes that play a variety of complementary roles within an ecosystem. Numerous studies have implicated the gut microbiota in health and disease in humans and in pets (1). Due to the high inter-individual variability of the gut microbiota, what constitutes healthy microbiota is hard to define and influenced by factors such as diet (2, 3), topographic location or environment (4, 5), genetics (6, 7), and more. However, the concept of microbiome resilience has recently gained attention and may be used as a marker of health (8). Resilience is the capacity of an ecosystem to recover from a modulating perturbation (9). A microbiota that is unable to recover from a perturbation may lead to a state of dysbiosis, negatively impacting the host and potentially contributing to the development of diseases like inflammatory bowel disease (10).

Although the microbiota is generally a stable community (11), perturbations like changes in diet, medication usage, and even psychological stress can lead to changes in its composition or function. For example, a diet change in dogs from a commercially available dry kibble diet to a highly purified diet led to significant changes in bacterial taxa and genetic potential within the community (12). However, after 36 weeks of being fed the highly purified diet, the microbiota quickly reverted back to its original state within 4–6 weeks of transitioning back to a dry kibble diet (12). Antibiotics are also a well-known stressor of the microbiome. In one study, dogs administered metronidazole for 2 weeks showed marked changes in their microbiota, with decreases in richness as well as in the relative abundance of Bacteroidetes and Fusobacteria and increases in Proteobacteria and Actinobacteria appearing after 7 days of treatment (13). In this study led by Pilla and colleagues, neither richness nor Fusobacteria relative abundance had fully recovered to pre-antibiotic levels 4 weeks post-treatment. Dogs undergoing transport-related stress also exhibited alterations in their fecal microbiota. Relative abundances of Actinobacteria, *Collinsella, Slackia, Ruminococcus*, and *Eubacterium* increased post-transport while relative abundances of Fusobacteria, *Streptococcus* and *Fusobacterium* were reduced (14, 15).

Physical exercise can also have an impact on the microbiota of dogs. Oba et al. compared microbiota changes in trained and untrained dogs subjected to exercise stress (15). In untrained dogs, the relative abundance of several bacterial genera was reduced, including *Bacteroides, Parabacteroides, Prevotella, Phascolarctobacterium, Fusobacterium*, and *Sutterella*, while *Collinsella, Slackia, Clostridium, Blautia, Ruminococcus, Megamonas, Catenibacterium* were increased. Interestingly, after training, dogs subjected to exercise challenge displayed significant changes in the relative abundance of even more fecal bacteria than untrained dogs. Of note, the relative abundance of *Turicibacter, Faecalibacterium*, and *Eubacterium*, genera with bacterial species known for their production of short chain fatty acids, increased post-exercise (15–17). Changes in the gut microbiota may also impact exercise performance. In endurance racing sled dogs, the teams with the best performances showed both the lowest levels of dysbiosis-associated bacteria [as measured by the canine dysbiosis index (18)] prior to the race and the lowest change (decrease) in these bacteria after the race (19).

Not only does exercise lead to changes in the gut microbiome, but it can also cause gastrointestinal symptoms, like diarrhea, in dogs. In racing sled dogs, this diarrhea does not seem to be correlated with the presence of pathogens (20). In attempt to overcome this, Gagné et al. tested a synbiotic treatment in sled dogs. Alterations in the fecal microbiome were observed with a significant rise in *Lactobacillaceae* in a group fed a synbiotic after 2 weeks of treatment. A positive correlation was found between *Lactobacillaceae* and overall butyrate concentration in all dogs. After 5 weeks of treatment, there was an improved fecal score and fewer days of diarrhea in the dogs given the synbiotic (21).

Recent studies unveiled that the stability of the microbiota is defined by two factors: first, the capability of the community to resist and recover from the constant disturbances arising in the environment, and second a high diversity dominated by competitive interactions among bacteria, leading to a rich complexity of metabolites that benefit the host (22). When the community is no longer resilient and incapable of overcoming stress, there is a rise of cooperative behaviors associated with a decrease in diversity and a potential reduction of the beneficial metabolic activity (23). For this reason, a system capable to withstand disturbances, remaining longer within the boundaries of the homoeostatic equilibrium, is a key requirement for a healthy relationship between host and microbiota (8). Understanding the mechanisms that promote resilience against the ever-occurring perturbations is important not only to understand the relationship between microbiota and health, but also to be able to promote the needed microbiome changes to lead toward health when disease occurs (24). To do so, we need methods to quantify the levels of resilience and the tools to manipulate it when needed.

The concept of resilience has been classically considered to be a dichotomic descriptive of a system (either it is resilient, or it is not). However, this view has been recently discarded toward a more quantitative perspective. As disturbances can be quantifiable (they have a magnitude and a length), so does the capacity of a system to withstand them and recover from them. Therefore, different methods have been developed to characterize and quantify resilience (8, 25). Such methods usually take two independent, yet complementary perspectives: the first one focuses on the length of the disturbance and the time taken to recover from it (henceforth recovery). The second one focuses on the magnitude of the disturbance. Such a change in perspective and its dual point of view has brought an understanding of the mechanisms underlying resilience. Yet, most of the methods to quantify resilience focus on one perspective or the other and do not account for the fact that resilience has this bivariate measurement in which both factors (impact and recovery time) influence the resilience of the community.

In this study we propose a new method to characterize microbiome resilience which accounts for both the impact of the disturbance and the recovery time. We apply this new method to sled dogs undergoing three types of exercise: running on an exercise wheel, pulling an all-terrain vehicle (ATV), and pulling a sled. The method is based on the multidimensional reductional approach of functional principal component analysis (FPCA). Our approach will help understand the relationship between gut microbiota and resilience and might help in disentangling the elements of the system affecting the impact of the disturbance and the recovery simultaneously.

## Methods

### Dogs and exercises

Fifteen healthy adult Alaskan husky dogs underwent three exercise types [running on the wheel, pulling an all-terrain vehicle (ATV), and pulling a sled] over a 6-month period. These dogs are very active by nature, with a natural ability to pull and a high desire to run. All exercises in the study are typically used for training in their sport. The dogs typically exercise 2 to 3 days per week, and samples were collected for this study on a normally scheduled exercise bout.

– Wheel: Dogs ran in a circle where a wheel spun at a fixed rate. Dogs were run in two groups on the same day in 7-dog and 8-dog teams. This exercise imitated a long, slow, low intensity run (14 miles total distance for 2-h time period averaging 7 miles/h). It was 55° F for this exercise. The wheel is around 40% VO_2_ max and is a non-pulling, aerobic exercise. The wheel builds the aerobic base of the dogs by getting over the 90-min threshold where glycogen stores become depleted and fat substrate use becomes more efficient and preferred. Building an aerobic base help support higher intensity training later in the season. This exercise was performed first in the series of exercises.
– ATV: Dogs ran on harness in front of an ATV for ~30 min for 7 miles at 14 miles/h. It was 20°F for this exercise. ATV pulling focuses more on strength training. This means that dogs pull more weight than they would on a sled, but the speeds are slower. This exercise was performed second in the series of exercises, 2 months after the wheel exercise samples were collected.
– Sled: Dogs ran on harness pulling a dog sled. Dogs ran a distance of 10 miles over a period of 40 min at ~15 miles/h. It was −7°F for this exercise. Sled pulling is the highest intensity of the three types of exercise. Dogs pull less because resistance is less, but they run at a faster pace. Dogs can push the 90% VO_2_ max level at this intensity. This exercise was performed last in the series of exercises, 4 months after the ATV exercise samples were collected.

The dogs in this study were fed a commercially available nutritionally complete and balanced dog food (Nestlé Purina product: ~29% protein, 36% carbohydrate, 19% fat; 1.4% fiber, 3894 ME Kcal/Kg). Dogs (8 female, 7 male) were between the ages of 1 and 7 years old and weighed between 40 and 65 pounds. Dogs were housed outdoors on elevated platforms in groups of four with access to an individual insulated house. The kennel is privately owned and located in Salcha, Alaska, USA. Exercises were performed by trained kennel staff using an exercise wheel in the kennel or in harness on a trail accessed directly from the kennel. All 15 dogs completed all three exercises, except for Dog 3, who only completed the ATV and Wheel exercises.

This study was approved by the Institutional Animal Care Use Committee at the University of Alaska Fairbanks (protocol # 1807962-1).

### Sample collections

Fecal samples or rectal swabs were collected at the following timepoints: Pre-exercise (feces), Post-exercise (rectal swab), 3 h post-exercise (rectal swab), 6 h post-exercise (rectal swab), 24 h post-exercise (feces), 48 h post-exercise (feces). Pre-exercise was collected the morning of the exercise before harnessing dogs to start exercise. Post-exercise samples were collected immediately at the end of the exercise bout. Naturally-eliminated fecal samples were collected within 30 min of defecation and frozen at −80°C. Rectal swabs were collected by a veterinarian with assistance from trained kennel staff and then stored at −80°C. All samples remained at −80°C until completion of study before being shipped on dry ice to Nestlé Purina for immediate sample processing and analysis.

Whole blood samples were collected at the same time points as fecal samples above (pre-exercise, post-exercise, 3 h post-exercise, 6 h post-exercise, 24 h post-exercise, and 48 h post-exercise), as approved in the study protocol by the University of Alaska Fairbanks Institutional Animal Care Use Committee (protocol # 1807962-1). Five milliliter of whole blood was collected from each dog by a veterinarian at each time point via cephalic venipuncture. Whole blood was put into tubes (BD Vacutainer SST tubes # 367988), left at room temperature for 1 h, then centrifuged for 10 min at 4,500 rpms. Serum was aliquoted into 500 μl aliquots and frozen at −80°C. Samples were shipped to on dry ice to Nestlé Purina for immediate sample processing and analysis.

### DNA extraction, 16s rRNA sequencing, and data processing

Fecal samples and swabs were extracted using Qiagen QiaAMP BiOstic Bacteremia DNA Isolation kit (catalog #12240-50). For fecal samples DNA extractions, ~0.2 g of feces was resuspended in 450 μl MBL Solution and transferred to the PowerBead Tube for extraction per manufacturer's instructions. For rectal swab DNA extractions, swabs are placed in 450 μl of MBL Solution and vortexed at max speed for 10 min. After vortexing, the swab is removed, and the lysate transferred to the PowerBead Tube for extraction per manufacturer's instructions.

Library preparation of DNA extracts was performed as described previously (26). DNA was quantified by Quant-It Pico Green (Fisher # P7589) and run on a 1% Agarose E-Gel (Fisher # G7008-01) with a 15 Kb high range ladder (Fisher # 12-352-019) to check for DNA integrity. Samples were normalized to 5 ng/μl using 10 mM Tris HCL, pH = 8.5 and re-quantified. PCR was performed using 12.5 μl 2X KAPA HiFi HotStart Ready Mix (Fisher # NC029523), 2.5 μl of the 5 ng/μl DNA, and 5 μl of each amplicon forward and reverse primers (1 μM each). PCR was performed as per 16S Metagenomic Sequencing Library Prep Protocol (available from Illumina website). The resulting PCR product was quantified by Pico Green to confirm amplification and a subset of samples were run on a Bioanalyzer DNA 1,000 Chip (Agilent # 5067-1504) for sizing (expected size 570 base pairs).

PCR products were cleaned using AMPure XP Beads (Fisher # NC9933872) and two 80% Ethanol washes as per manufacturer protocol. The plates were then air dried and 55 μl of Tris HCl is added to each samples. The plates were placed on the magnetic stand and 50 μl of the cleared supernatant was transferred to a clean PCR Plate. An Index PCR was run to barcode the individual samples. Five microliter of the first PCR product for each sample was added to a master mix containing 25 μl of 2X KAPA HiFi HotStart Ready Mix, and 10 μl of Molecular grade water (Fisher # BP2819-1). Five microliter Nextera XT Index Primer 1 (N7xx) and 5 μl Nextera XT Index Primer 2 (S5xx) were added to the samples, so that each sample has a different and unique combination of the two primers. The PCR thermocycler conditions were 95°C for 3 min, 8 Cycles of 95°C for 30 s, 55°C for 30 s, 72°C for 30 s, and a final extension of 72°C for 5 min, hold at 4°C. The Index PCR products were cleaned as above except 30 μl Tris HCl was added to each samples. The plates were placed back on the magnetic and 25 μl of the cleared supernatant was transferred to a clean PCR Plate. Pico Green of the PCR Product was used for quantification and to confirm amplification. A subset of samples were run on a Bioanalyzer DNA 1,000 Chip for sizing (expected size 630–650 base pairs).

Each sample was then normalized to 20 nM using Tris HCl and 3 μl of each sample were combined to give a 20 nM Pool (Library). The 20 nM Pool was quantified by KAPA qPCR (Fisher # NC0833039). The Pool was diluted and 4 μl of the dilutions were added to a master mix containing 2X SYBER Fast with Primer Premix (From KAPA Kit) and Molecular grade water and run against the six standards from the kit (Standard Curve). After the run, a melt curve analysis was performed on the samples. Expected melt temperature is around 85°C. Using the concentration from the qPCR, the pool is diluted down to 4 nm with Tris HCl and a KAPA qPCR is run again to confirm 4 nM Pool.

PhiX control was diluted to 4 nM with Tris HCL and 0.1% Tween 20. Both PhiX control and the Pool were denatured with 0.2 N NaOH, and diluted to 20 pM using HT-1. Both the 20 pM DNA and 20 pM PhiX control were diluted again to a final loading concentration of 13 pM. Pool and PhiX control were combined to yield 14% PhiX spike. The V3-V4 region of the 16S rRNA was sequenced on an Illumina MiSeq using a MiSeq Reagent kit v2 (500 cycles; Illumina catalog # MS-102-2003). Reads were de-multiplexed and paired, and primer sequences were removed by trimming the first 17 bases of the forward reads and 21 bases from the reverse reads. The DADA2 algorithm, implemented in QIIME2 (version 2021.4), was used to denoise the data and identify and quantify counts of amplicon sequence variants [ASVs; (27, 28)]. A naïve-Bayes classifier trained on the Silva 138.1 SSU Ref NR 99 database was used to assign taxonomy to each ASV.

### Inflammatory markers

Inflammatory markers were measured in serum using Millipore Milliplex MAP Canine Cytokine/Chemokine Premixed Magnetic Bead Kit (catalog # CCYTMG-90K-PX13) according to the manufacturer's instructions on a Luminex 200 (Thermo Fisher Scientific catalog # APX10031). Antibody-coated detection beads were incubated overnight at 4°C with appropriate standards, samples, or quality controls in 96-well plates. Serum sample volume of 25 μl (undiluted) was used. Recombinant cytokines were provided in the kit to serve as standards. All standards, samples, and controls were run in duplicate. The following cytokines and chemokines were measured at each timepoint: Granulocyte-macrophage colony-stimulating factor (GM_CSF); interleukins 6, 7, 8, 10, 15, 18, interferon-gamma inducible protein-10 (IP-10), keratinocyte chemoattractant-like (KC-like), monocyte chemoattractant protein-1 (MCP-1), and tumor necrosis factor-alpha (TNF-alpha). Luminex data was analyzed using the Luminex xPONENT software (version 3.1 Build 971) to determine concentrations of each cytokine/chemokine. All standards and controls were within the expected concentration ranges. Missing values indicate that the cytokine/chemokine was below the limit of detection for the assay.

### Measuring resilience

Our approach consisted in describing the evolution of the microbial community by analyzing the trajectories that summarize the magnitude of change at each timepoint.

The calculation of the trajectories requires the choice of a reference time point, and a choice of a distance between two compositions (by composition, we mean the percentages of relative abundance of the taxa under consideration). In this study, we chose the pre-exercise time point as reference time point. The Aitchison distance was chosen as a metric to measure the amount of change of the microbiome composition between two time points. The Aitchison distance between two compositions is defined as:

$$\begin{array}{l} d\left(u,v\right)={\left[\overset{D}{\underset{k=1}{\sum }}{\left(\log\left(\frac{u_{k}}{g\left(u\right)}\right)-log\left(\frac{v_{k}}{g\left(v\right)}\right)\right)}^{2}\right]}^{1/2} \end{array}$$

where *u* = (*u*_1_, …, *u*_*D*_), *v* = (*v*_1_, …, *v*_*D*_) are two vectors representing the relative abundances of D taxa in two samples. The components of these vectors are non-negative numbers, whose sum is 1. The *g*(*u*) term is the geometric mean of the vector *u*. The formula above defines a mathematical distance between compositions, which is consistent with respect to feature selection. This property is not shared by other commonly used measures, including the Jensen-Shannon divergence. On the other hand, the formula requires all the relative abundances to be non-zero, therefore we applied the zero-replacement method *cmultRepl* from the R package *zCompositions* as a pre-processing step before calculating the distances.

Microbiome data is often high-dimensional: the number of bacteria can be bigger than the number of samples. To address the high-dimensionality and sparsity of the data, we applied log-ratio analysis (LRA) to select a list of taxa from the full list, for further use in the modeling. Our assumption was that the essential information in the data lies mostly in fewer dimensions. This assumption is supported by the observation that most of the species, or genera, have a measured relative abundance of 0% for almost all the dogs at almost all time points, with no discernible pattern regarding their sparsity. Log-ratio analysis is equivalent to a weighted principal component analysis on the center-log-ratios of the data, with compositional part means as weights. It allowed us to rank the variables according to their contributions to the total variance, and we selected in each dataset the minimum number of taxa explaining at least 90% of the variance.

Having calculated all the distances with respect to the pre-exercise point, we were able to trace a microbiome trajectory for each dog and each exercise type. The next step was to define a list of parameters, minimal in some sense, describing the common patterns in the family of trajectories. Like PCA, Functional Principal Component Analysis (FPCA) aims at finding key features of the data that explain a maximum of variability; however, while PCA applies to cross-sectional data, FPCA is an algorithm specifically designed for longitudinal data. The algorithm follows the general paradigm of functional data analysis, whereby time-dependent measurements are seen as samples from smooth curves. If *X*_*i*_(*t*) represents the value of the trajectory of individual *i* at time *t*, we can write its Karhunen-Loève decomposition:

$$\begin{array}{l} X_{i}\left(t\right)=\mu \left(t\right)+\sum_{k=1}^{\infty }\xi_{k}\phi_{k}\left(t\right) \end{array}$$

where μ(*t*) is the population average at time *t*, and ϕ_*k*_ are the eigenfunctions of the covariance operator.

The FPCA scores are defined as:

$$\begin{array}{l} \xi_{k}=\int \left(X_{i}\left(t\right)-\mu \left(t\right)\right)\phi_{k}\left(t\right)dt \end{array}$$

In practice, one truncates the above infinite sum to the first N terms, the higher the N the higher the proportion of variability explained by the sum. Empirically, we found that the first 2 terms suffice to explain more than 90% of the variance in all the cases we considered.

The first two FPCA scores can then be used to summarize our data: to each trajectory we attached the pair (FPCA score 1, FPCA score 2), and we related these two numbers to the degree of resilience.

The FPCA framework can also be used to identify outliers in the family of trajectories. The approach consists of creating a set of convex hulls of the scores based on the bagplot method, a bivariate generalization of the univariate boxplot. This approach helps to detect trajectories whose shape is globally ‘atypical' in the population, in a data-driven way, as opposed to defining outliers based on an a-priori choice of a single feature of the curves (e.g., area under the curve).

As a way to better understand the interpretation and potential of our proposed approach, we simulated families of trajectories by letting the 2 scores vary independently on a regular grid of values. This gives a clear picture of how the scores jointly describe the shapes of trajectories, and how this may be interpreted in terms of resilience.

## Results

Typically, relative abundances of bacteria in the gut are unbalanced, with few bacteria being present in almost all samples, and many others being absent from almost all samples. The genus Mycoplasma, for example, was detected only in five samples out of 263, with a maximal relative abundance of 3.3%. On the other hand, *Peptoclostridium, Fusobacterium, Fecalibacterium, Blautia, Bacteroides, Allobaculum* were always present, with an average abundance of, respectively, 11.7%, 12.9%, 3.2%, 5.2%, 7.2%, 2.0%.

From the compositional bar plots (Supplementary Figures 1–3) we observed some general features of the data: in the Sled and ATV data, *Lactobacillus* was highly abundant in the gut microbiota of several dogs, and nearly absent in others, *Bifidobacterium* was highly abundant only in the Sled data, while *Blautia* and *Peptoclostridium* had similar values and patterns of change in the three exercise datasets.

**Figure 1:**
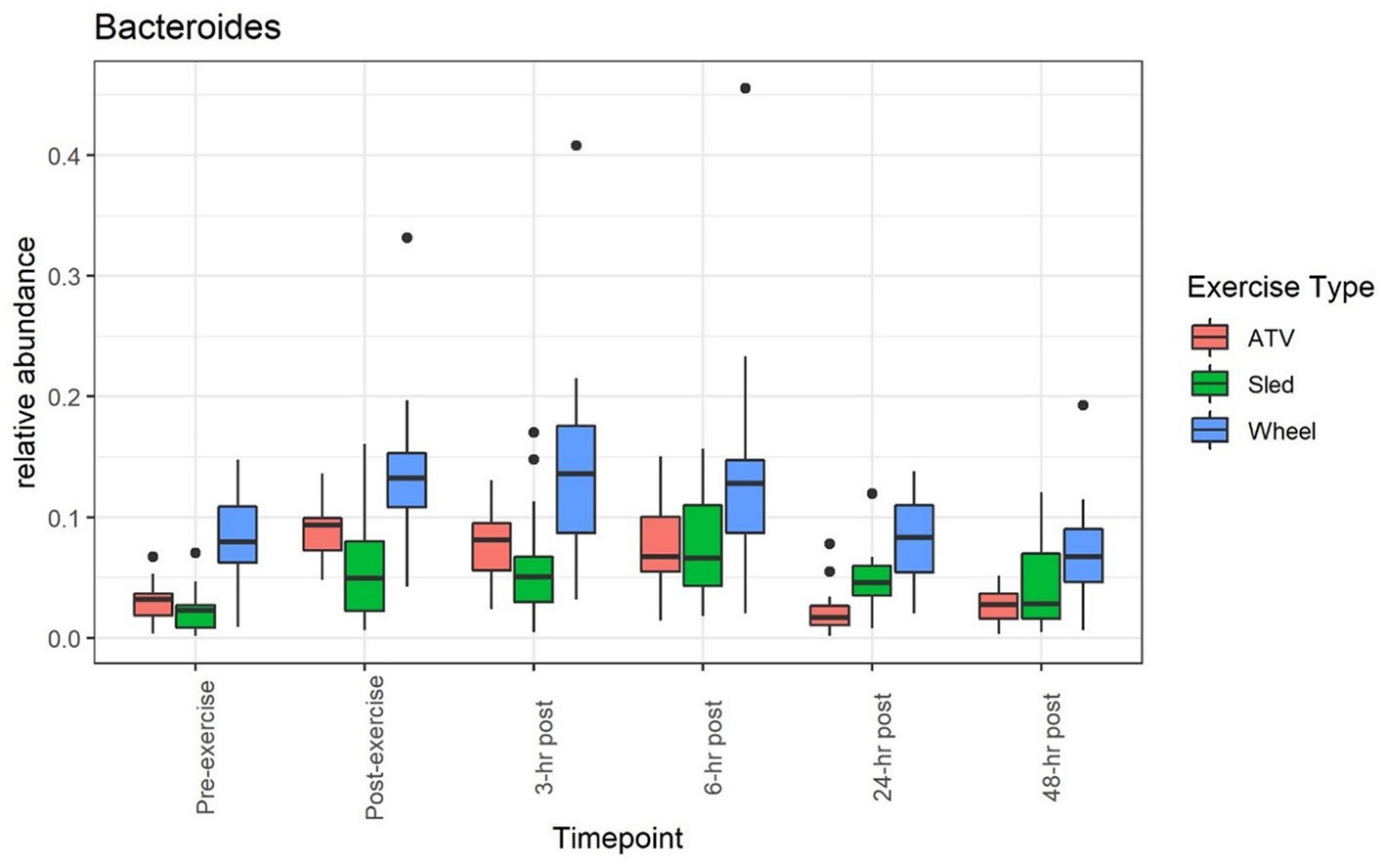
Relative abundance of *Bacteroides* increases during and after the exercise, and returns to values close to the pre-exercise levels afterwards.

**Figure 2:**
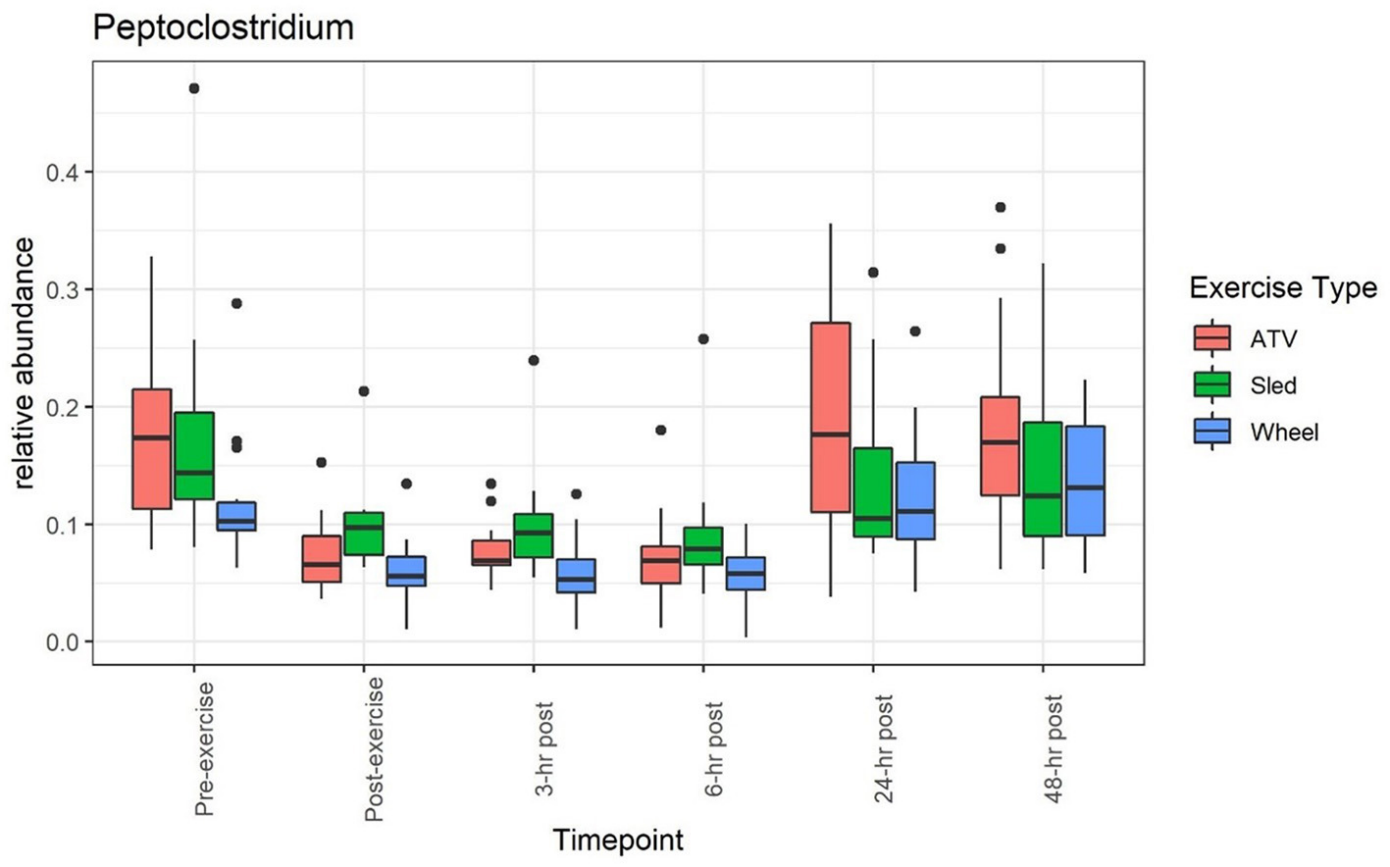
Relative abundance of *Peptoclostridium* decreased during and after the exercise, and returned to values close to the pre-exercise levels afterwards.

A clear impact of voluntary exercise stress can be observed for the relative abundance of several bacteria when taken individually (see, for example, Figures 1, 2): the average abundance of *Bacteroides* increased from 8.3 to 14.4% before and after the wheel exercise, while the relative abundance of *Peptoclostridium* decreased from 12 to 6.1%. However, for other taxa (e.g., *Bifidobacterium*, Supplementary Figure 4 and *Prevotella*, Supplementary Figure 5) we did not observe a significant time trend. Supplementary Table 1 provides a list of statistical comparisons of the relative abundance, before and 3 h after the exercise, for the main taxa.

**Figure 3:**
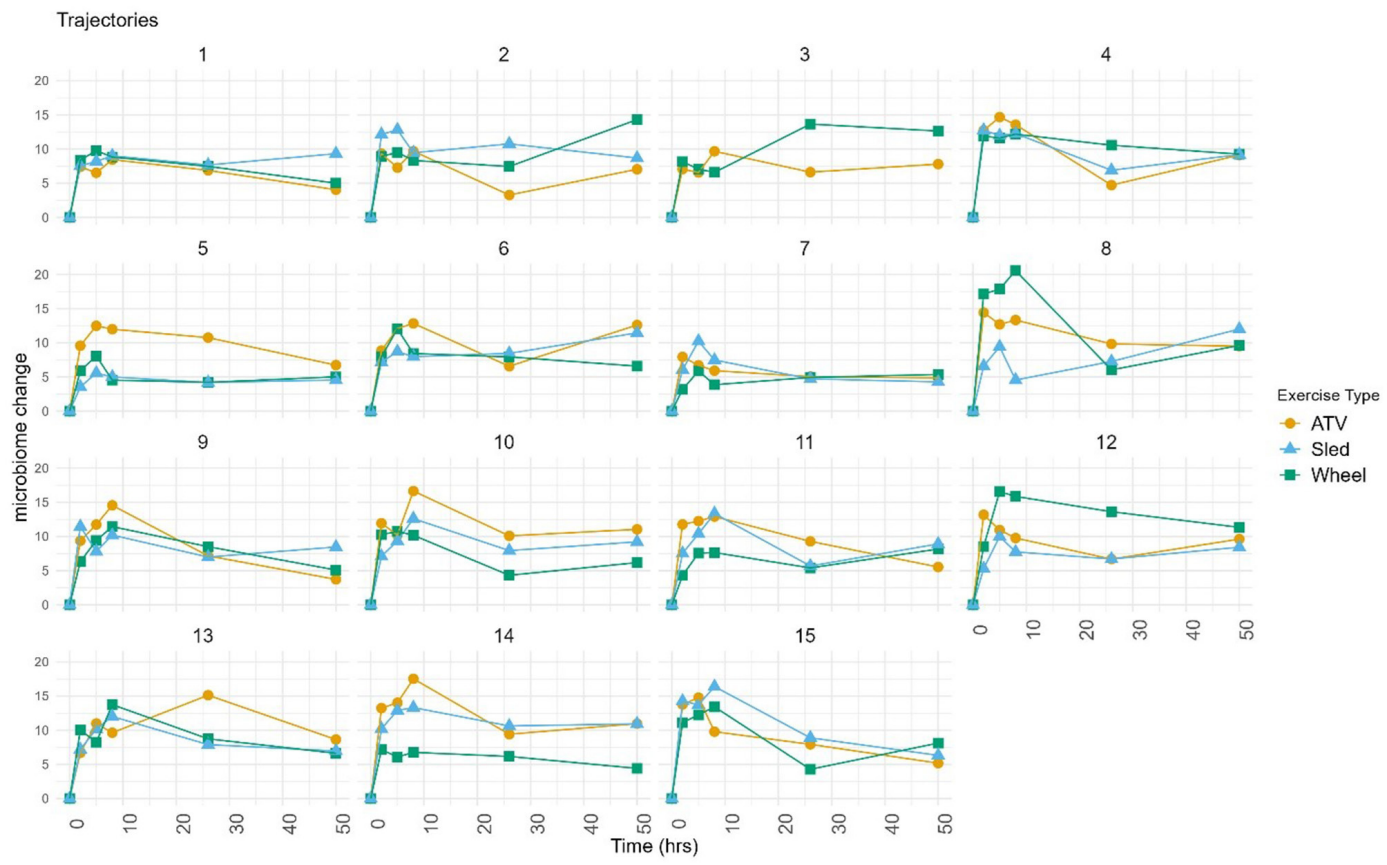
Trajectories calculated on selected variables, with respect to the pre-exercise timepoint. Each point corresponds to the (Aitchison) distance between the composition at the given timepoint and at pre-exercise. Each chart shows the trajectories of a same dog corresponding to the three exercise types. Dog three did not complete the sled exercise.

**Figure 4:**
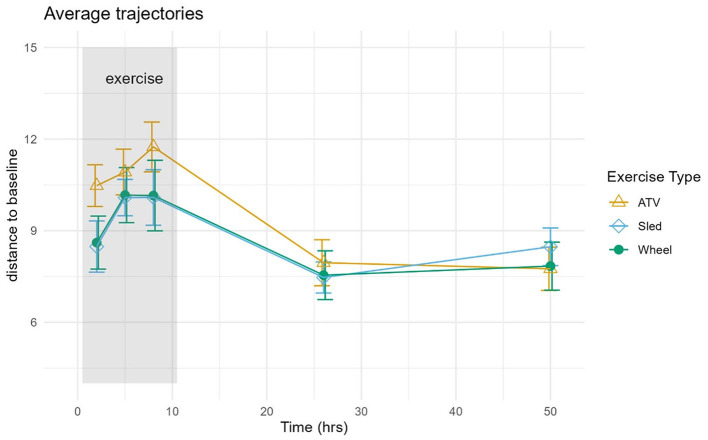
The average distance and standard error were calculated for each exercise type separately and plotted as functions of time in hours.

Log-ratio analysis for dimensionality reduction identified 33 variables explaining 90% of the variance (Table 1). *Lactobacillus* was ranked highest, followed by *Prevotella* and *Corynebacterium*. Inspection of the trajectories (Figure 3) suggests that the microbiome composition changed significantly during the 6 h following the exercise, with a partial stabilization/recovery afterwards. The average trajectory suggested a stronger perturbation due to the ATV training, with no noticeable differences in the recovery phase (Figure 4).

**Table 1:** Variables selected by log-ratio analysis, ordered by decreasing proportion of explained variance.

**Genus**	**Contribution**	**Genus**	**Contribution**
[1] *Lactobacillus*	13.6%	[18] *Blautia*	1.7%
[2] *Prevotella*_9	9.3%	[19] *Turicibacter*	1.5%
[3] *Corynebacterium*	7.3%	[20] *Allobaculum*	1.4%
[4] *Catenibacterium*	4.6%	[21] *Eubacterium*_brachy_group	1.4%
[5] *Peptoclostridium*	4.4%	[22] *Escherichia*-*Shigella*	1.3%
[6] *Bacteroides*	4.2%	[23] *Faecalibacterium*	1.3%
[7] *Limosilactobacillus*	4.1%	[24] *Ralstonia*	1.3%
[8] *Alloprevotella*	3.9%	[25] *Erysipelotrichaceae* genus uncultured	1.3%
[9] *Ligilactobacillus*	3.8%	[26] *Streptococcus*	1.1%
[10] *Peptostreptococcus*	3.2%	[27] *Peptococcus*	1.0%
[11] *Prevotellaceae*_Ga6A1_group	2.3%	[28] *Sutterella*	1.0%
[12] *Muribaculaceae*	2.1%	[29] *Phascolarctobacterium*	0.8%
[13] *Clostridium*_sensu_stricto_1	2.1%	[30] *Dubosiella*	0.8%
[14] *Fusobacterium*	1.9%	[31] *Erysipelatoclostridium*	0.8%
[15] *Parasutterella*	1.8%	[32] *Ruminococcus*_torques_group	0.7%
[16] *Anaerobiospirillum*	1.8%	[33] *Lachnospiraceae* (genus unknown)	0.7%
[17] *Bifidobacterium*	1.7%		

**Figure 5:**
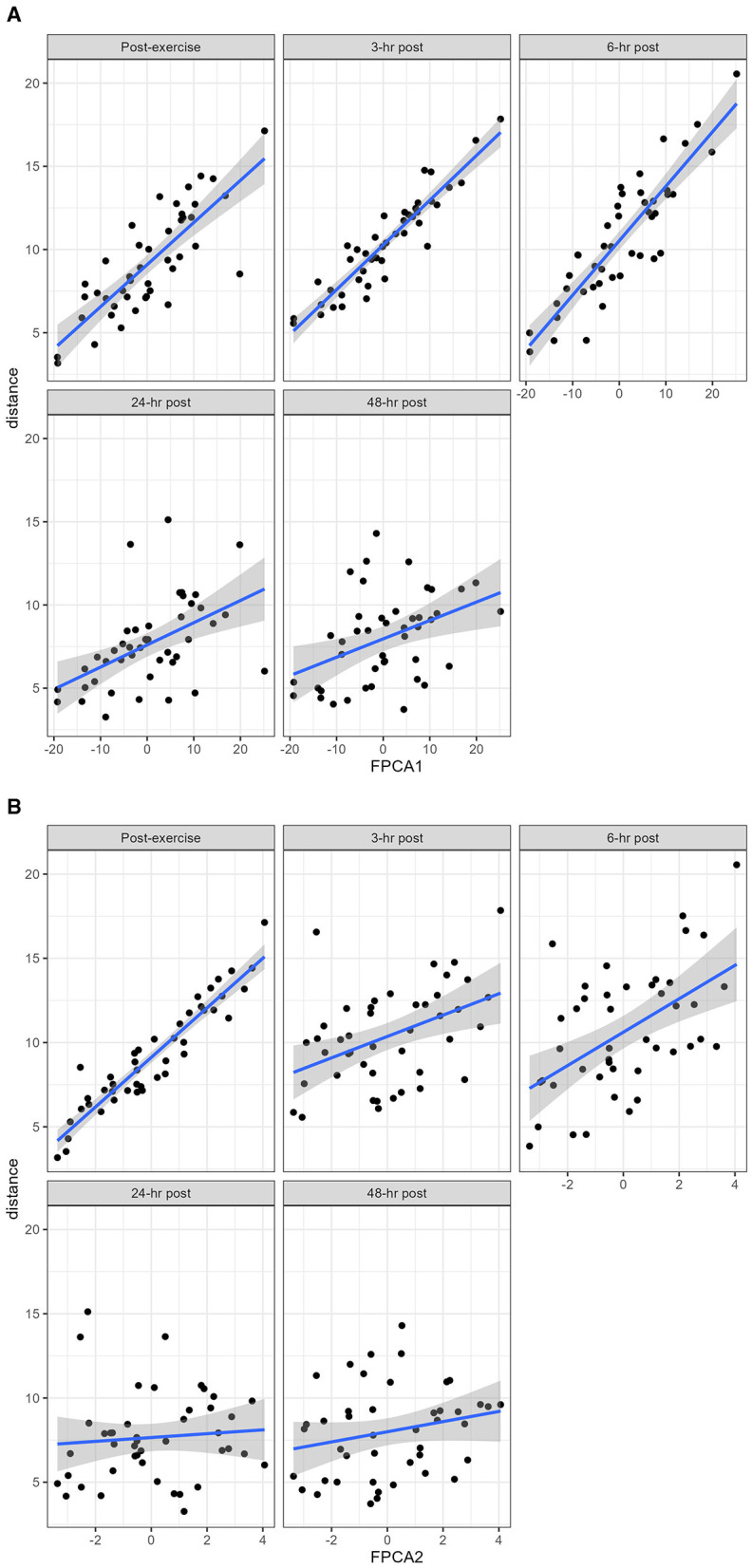
The distance at each timepoint was plotted against the FPCA scores. **(A)** the first score is strongly positively correlated with the distance at all timepoints; **(B)** the second score is negatively correlated with the distance until 6 h post exercise, there is then an inflection point between 6 and 24 h where the correlation becomes positive.

**Figure 6:**
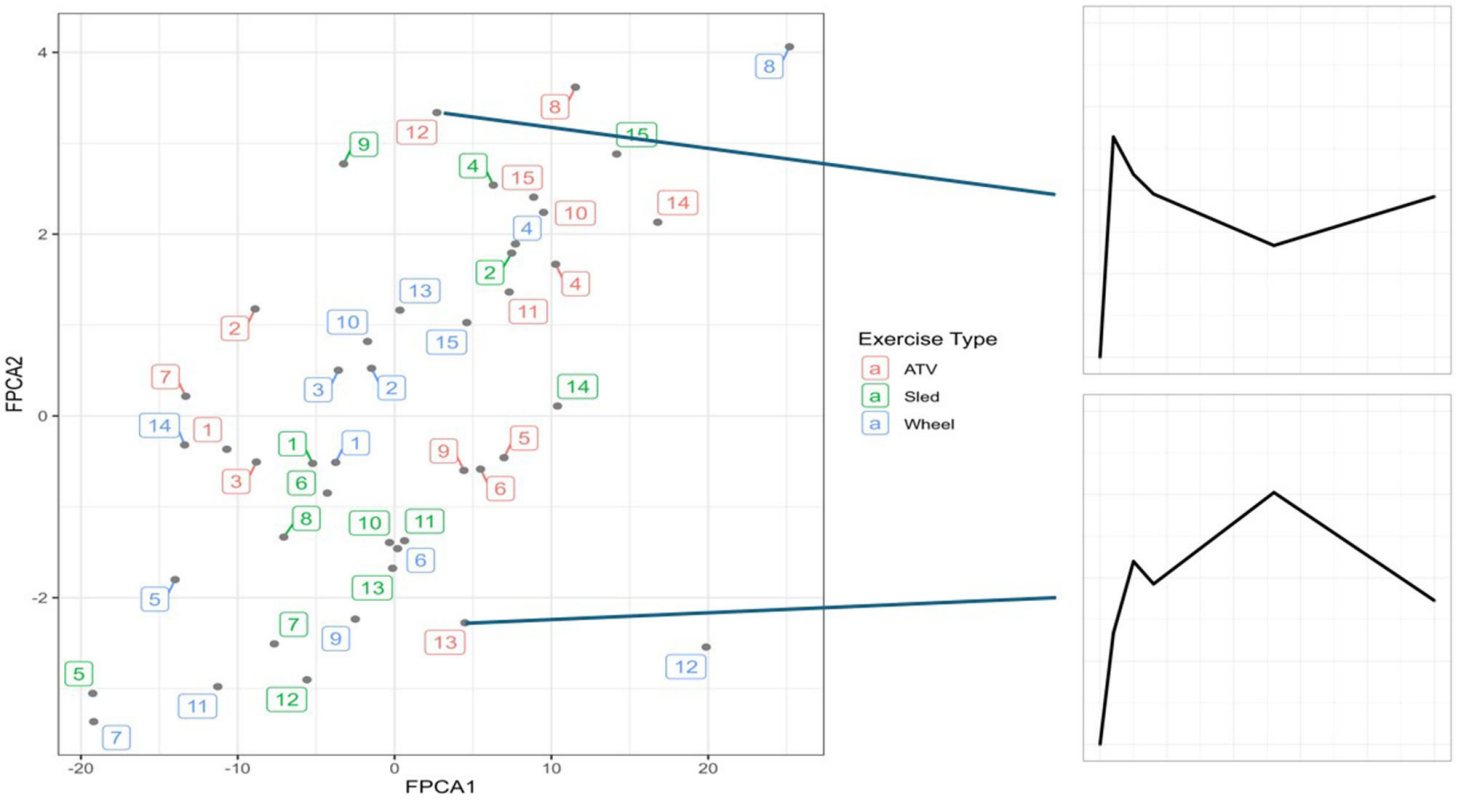
The two scores plotted together, with each point corresponding to a trajectory. On the right side, two examples of outlier trajectories, at the two extremes of the FPCA2 range. Numbers represent dogs 1–15, colors represent exercise type.

FPCA identified a first score explaining 76% of the variability of the trajectories, and a second score explaining 19% of the variability. The interpretation of the 2 scores can be further understood from the observation that the first score was positively correlated with the distance at all timepoints, and the second score was negatively correlated with the distance until 6 h post-exercise, and positively correlated afterwards (Figure 5). A scatterplot of the two scores (Figure 6) helps to identify trajectories that share a similar shape, because they correspond to points that are close to each other, as well as outliers.

**Figure 7:**
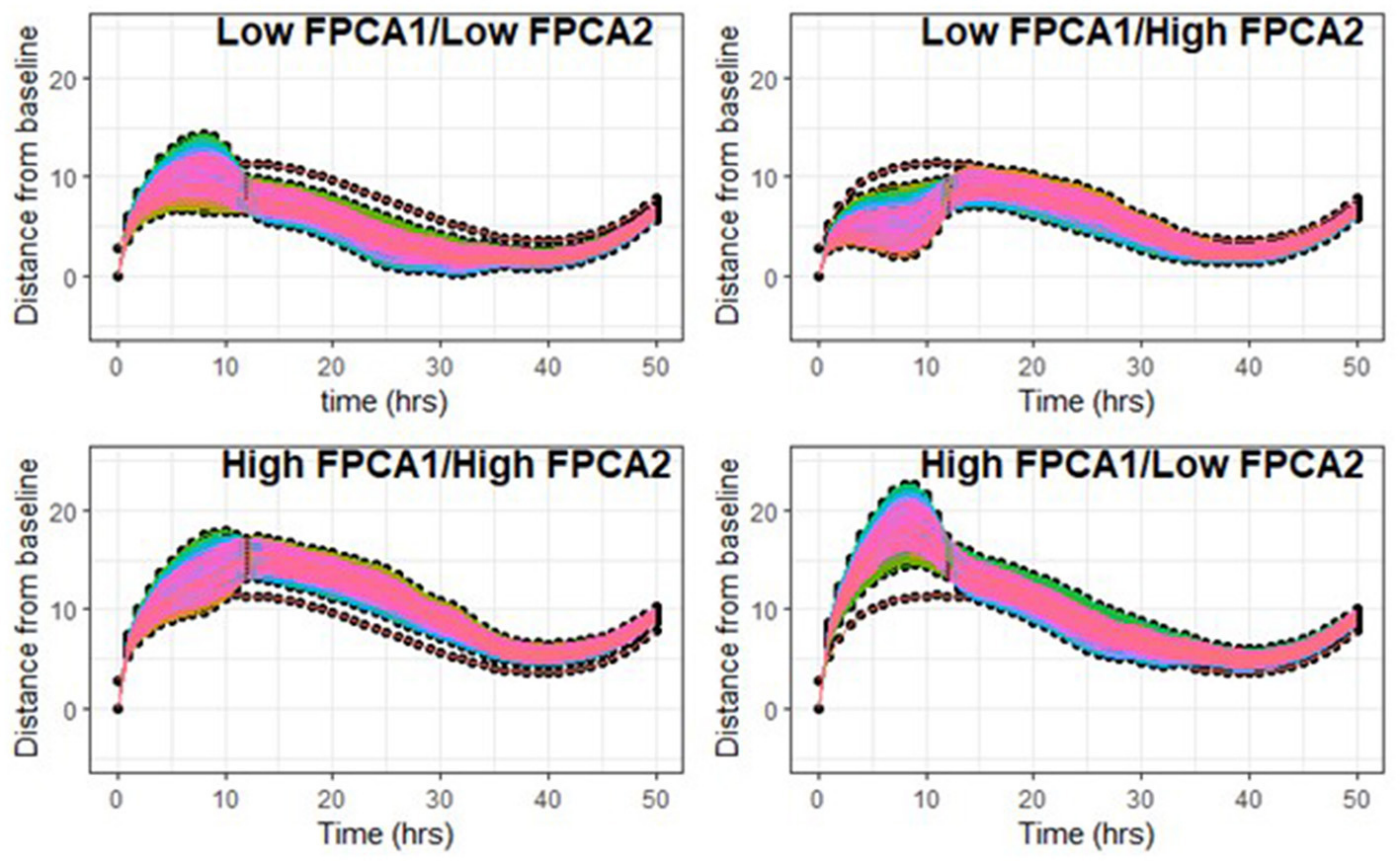
Top left: FPCA1 scores vary in the first quartile [−25, −6], FPCA2 scores in the first quantile [−16, −4]. Top right: FPCA1 in [−25, −6], FPCA2 scores in the highest quartile [1, 11]. Bottom left: FPCA1 in the highest quartile [9, 30], FPCA2 scores in [1, 11]. Bottom right: FPCA1 in [9, 30], FPCA2 in [−16, −4].

We simulated families of trajectories by letting the two scores vary independently on a regular grid of values (Figure 7). Figure 7 supports the fact that the shapes of trajectories could not be accurately described with only one parameter, and that both scores are needed in combination to capture the complex patterns of variability.

More resilient trajectories correspond to low (negative) values of FPCA1 and values of FPCA2 close to 0 (compare with trajectories of Dog 5-Sled and Wheel, Figures 3, 6). Although FPCA1 was generally higher in the ATV group (4.4 ± 11.3, 0.2 ± 10.0 for Sled and 0.2 ± 14.3 for Wheel), the difference was not significant. Values of FPCA2 were −2.3 ± 4.2 (ATV), 0.0 ± 4.3 (Sled), −1.5 ± 6.5 (Wheel), also not significant.

Each dog (except Dog 3) had three trajectories in this dataset, corresponding to the three exercise types, performed at different times of the year. For each exercise challenge, each trajectory was calculated using the pre-exercise timepoint as baseline. We calculated again the trajectories using as baseline the pre-exercise timepoint before the wheel exercise, which occurred first chronologically. The mean distance from baseline was 10.4 ± 0.48. By comparison, the average distance when using different baselines was 6.2 ± 0.28, sensibly lower. Visual inspection of the trajectories suggests a significant change of the pre-exercise time point in the long term (Supplementary Figure 6).

**Figure 8:**
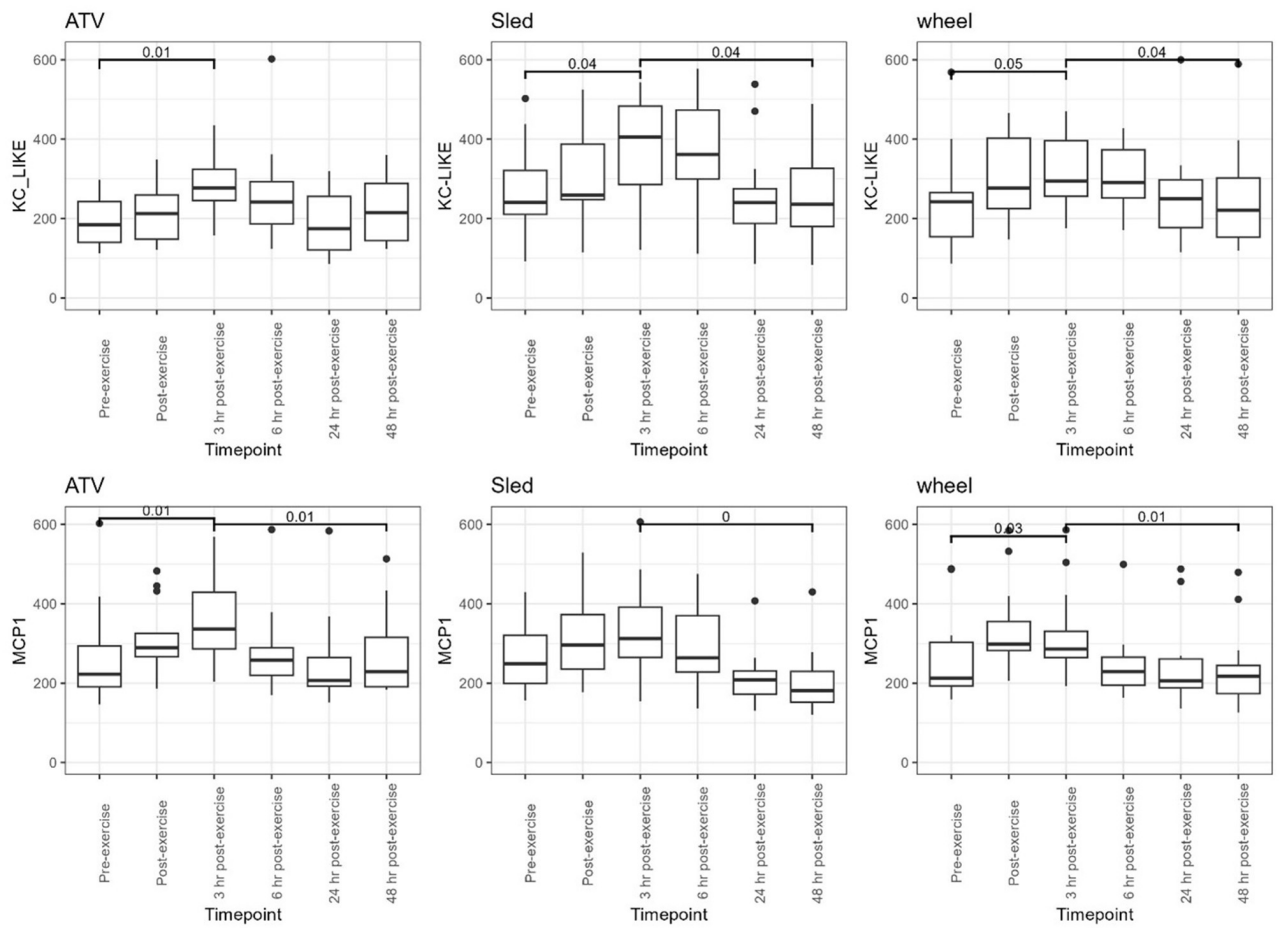
Distributions of KC-like and MCP-1, for each of the exercise types, are shown as boxplots for every time point. Above the boxplots, *p*-values are shown to compare the distributions between pre-exercise and 3 h post exercise, and between 3 h post and 48 h post-exercise. Dunn *post-hoc* tests with FDR correction were used for comparison.

**Figure 9:**
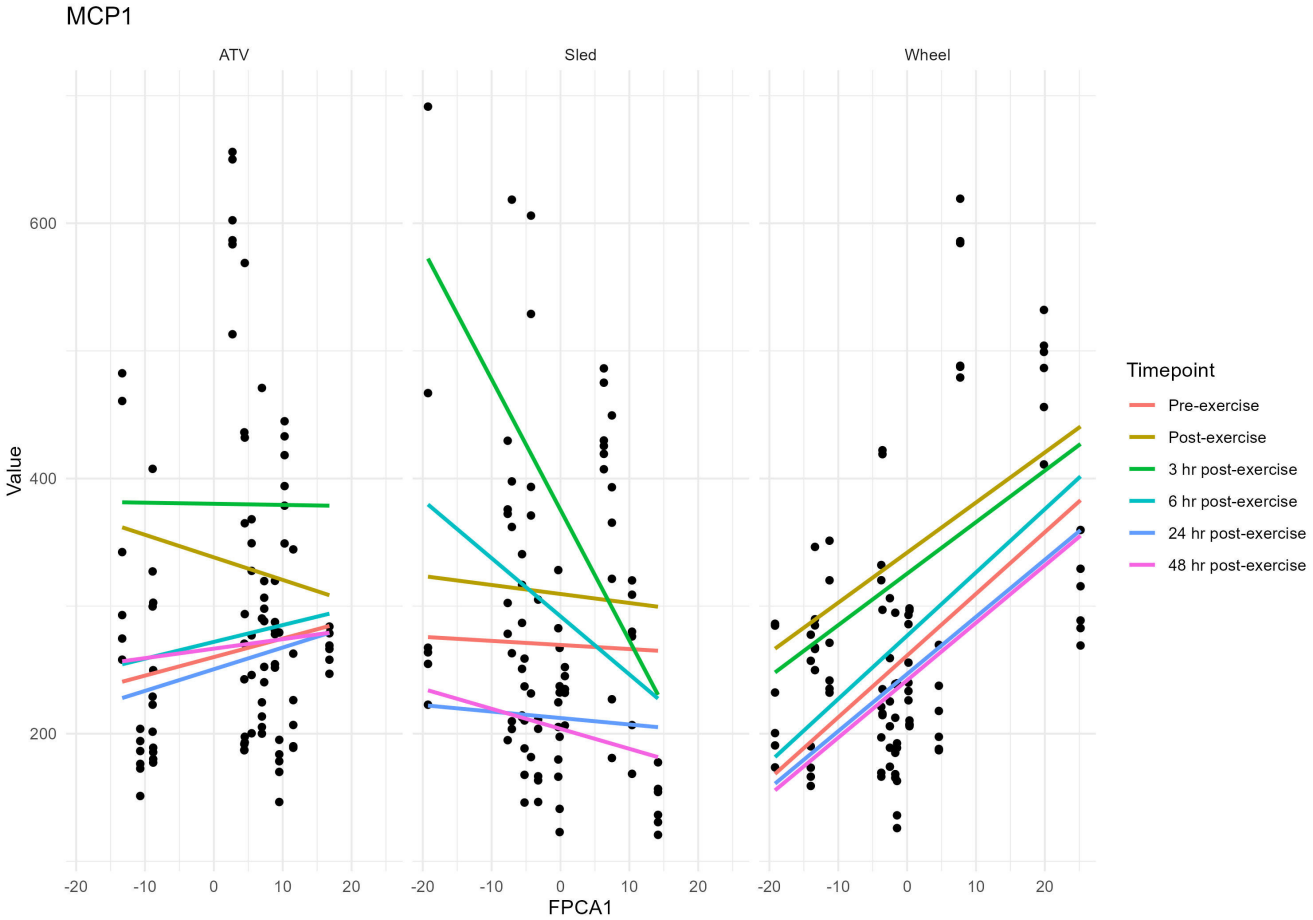
Values of MCP-1 plotted against values of FPCA1. Each dot corresponds to a sample.

Levels of chemokines MCP-1 and KC-like were impacted by the exercise, irrespective of the type of exercise (Figure 8). The levels of the remaining measured inflammatory markers were either not impacted by the exercise, or the effect was not consistent across the exercise types (see Supplementary Table 2 for descriptive statistics). However, for some of the cytokines and chemokines, for instance TNF-a, the proportion of missing values (due to low levels of these markers in serum) was very high, it is conceivable that this may have had an influence on the lack of association. For KC-like and MCP-1, our dataset was complete. FPCA scores were associated with the levels of the chemokines MCP-1 (Figure 9). A mixed linear model with random intercepts was fitted to the data, with the chemokine levels as outcome and the FPCA scores as predictors, together with interaction terms to model the theoretical effect of the exercise type. KC-like levels were significantly higher in the Sled group (Table 2) and were associated with FPCA1 only through exercise type. MCP-1 levels were lower in the sled group (Table 3) and were positively associated with FPCA2.

**Table 2:** Summary table for the linear mixed model KC_like ~ FPCA1 + FPCA2 + FPCA1^*^Exercise_Type + FPCA2^*^Exercise_Type + (1|ID).

	**KC-LIKE**		
**Predictors**	**Estimates**	***CI***	***p***
(Intercept)	259.12	195.97 to 322.27	**<0.001**
Exercise type [Sled]	44.42	16.58 to 72.26	**0.002**
Exercise type [Wheel]	30.96	4.57 to 57.34	**0.022**
FPCA1	1.28	−0.62 to 3.18	0.187
FPCA2	4.26	−0.60 to 9.13	0.086
Exercise type [Sled] ^*^FPCA1	−3.78	−6.35to −1.21	**0.004**
Exercise type [Wheel] ^*^FPCA1	−0.52	−2.88 to 1.84	0.664
Exercise type [Sled] ^*^FPCA2	1.94	−4.71 to 8.59	0.566
Exercise type [Wheel] ^*^FPCA2	−4.91	−10.58 to 0.75	0.089
**Random effects**			
σ^2^	5679.85		
τ_00__Dog_	13626.38		
ICC	0.71		
N _Dog_	15		
Observations	264		
Marginal *R*^2^/conditional *R*^2^	0.055/0.722		

Values in bold indicate significant *p*-values. Asterisks indicate interaction between two terms.

**Table 3:** Summary table for the linear mixed model MCP-1 ~ FPCA1 + FPCA2 + FPCA1^*^Exercise_Type + FPCA2^*^Exercise_Type + (1|ID).

	**MCP1**		
**Predictors**	**Estimates**	***CI***	***p***
(Intercept)	315.38	271.30 to 359.45	**<0.001**
Exercise type [Sled]	−38.09	−69.66 to −6.53	**0.018**
Exercise type [Wheel]	−36.67	−66.77 to −6.58	**0.017**
FPCA1	−1.05	−3.17 to 1.08	0.332
FPCA2	6.12	0.63 to 11.62	**0.029**
Exercise type [Sled] ^*^FPCA1	−0.58	−3.50 to 2.33	0.694
Exercise type [Wheel] ^*^FPCA1	4.10	1.45 to 6.74	**0.003**
Exercise type [Sled] ^*^FPCA2	3.40	−4.12 to 10.91	0.374
Exercise type [Wheel] ^*^FPCA2	−4.92	−11.32 to 1.49	0.132
**Random effects**			
σ^2^	7478.31		
τ_00__Dog_	5203.23		
ICC	0.41		
N _Dog_	15		
Observations	264		
Marginal *R*^2^/Conditional *R*^2^	0.112/0.477		

Values in bold indicate significant *p*-values. Asterisks indicate interaction between two terms.

## Discussion

Microbiota stability is an important ecological feature as it might impact the homeostasis of the host. Loss of homoeostasis may lead to dysbiosis and affect the host either locally in the gastrointestinal tract or systemically through its interaction with the immune system (1). Hence, assessing the levels of stability of the gut microbiota, and its resilience to environmental disturbances is a key question that might depend of the nature of the disturbance.

Consistent with findings reported by other authors (15), we observed a change in the composition of the gut microbiota after intense physical exercise in a population of trained Alaskan sled dogs. The intensity of this change reached a peak between 3 and 6 h after the exercise. These observations make the exploration of intense physical exercise as a potential disturbance appropriate.

Another open question is the methodology to quantify resilience in the ecosystem. The choice of a metric determines the way different trajectories are labeled as ‘resilient'. Indices that have been previously proposed (25) translate a prior view of which characteristics should be encoded in the definition of resilience. Typically, proposed indices measured resilience with one number, derived from the choice of some salient features of the trajectory, for example a slope between two points. Depending on this choice, these metrics try to capture either the resistance or the recovery of the ecosystem. A few studies have jointly considered multiple attributes of resilience (25, 29). It was already identified (25) that recovery time, or perturbation, does not provide a complete and sufficient description of the resilience of an ecosystem. In line with the bivariate framework proposed by Ingrish and Bahn (25), our proposed data-driven approach suggests an intrinsic 2-dimensional parametrization of the family of trajectories describing the response to a perturbation. The application of functional PCA aims to describe the trajectories with a limited number of parameters, or scores, each corresponding to a mode of variation with respect to the mean. This dual nature of resilience is confirmed in the exercise study, where the first 2 scores explained 95% of the variability.

Due to the high-dimensionality and sparsity of the microbial composition, we applied a data-driven technique to select a smaller list of taxa to be used for the analysis. This selection is dataset-specific and is based on the variability of the taxa, independently on their average abundance; in other words, the process does not favor highly abundant taxa, but low abundance bacteria can also be selected, as long as they contribute significantly to the total variance in the data. An alternative approach might consist in an *a priori* choice of bacteria that are known to be affected by the stressor, based on prior knowledge. In our case, little was known about the effect of physical exercise on the gut microbiome. Gagné et al. (21) have investigated the alterations in fecal quality, short-chain fatty acids, and the fecal microbiome in two groups of training sled dogs fed a synbiotic or microcrystalline cellulose placebo, highlighting the role of *Lactobacillus*. Interestingly, *Lactobacillus* was also selected by our procedure, and was ranked highest, increasing significantly after the exercise from 2 to 5–6%. *Prevotella_9, Bacteroides* and *Peptoclostridium* were also ranked as top contributors. It is known that *Prevotella_9* and *Bacteroides* are highly variable between dogs (30). In our study, the relative abundance of *Bacteroides* increased significantly after the exercise and recovered afterward. The relative abundance of the genus *Blautia* decreased significantly between pre- and post-exercise. In a previous study, *Blautia* was found to be more abundant in healthy dogs, compared to dogs with chronic enteropathy (18).

The quantification of resilience also depends on a reference composition, considered as a stable state with respect to which the successive states are compared. In our exercise data, we observed a significant variability in the pre-exercise state compared to the change induced by the physical exercise. The high degree of variability of the pre-exercise timepoint between exercises could reflect seasonal oscillations. It has been demonstrated that the gut microbiota is impacted by temperature. The gut microbiota of mice exposed to extreme cold (−5°C) and extreme heat (35°C) for 2 months differed significantly from those living at room temperature [25°C; (31)]. Indeed, outside temperature for both the ATV and sled exercises was below freezing. Therefore, outside temperatures could be impacting the variability in the microbiota that we observed within each dog in the pre-exercise time point and how the microbiota responds to the exercise. However, in the current study, weather and ground conditions limited when each exercise could be completed.

In this study, rectal swabs were utilized in order to capture the changes to the microbiota at distinct time points after the exercises. Although the reference composition (pre-exercise sample) was determined by a fecal sample, not a rectal swab, several human studies have shown that the fecal microbiota are comparable to the communities obtained from a rectal swab (32–34). While this comparison has yet to be studied in canines, a comparison of piglet fecal microbiota samples and rectal swab samples also showed that samples clustered by individual and not sample type (35). While utilizing the same sample type throughout the longitudinal sampling is preferred, waiting for natural defecation by the sled dogs would have likely resulted in missing the full magnitude of the disturbance due to exercise.

The chemokines KC-like and MCP-1 increased significantly after the exercise and returned to levels comparable to pre-exercise after 24 h, providing further evidence of a significant physiological stress imposed by the intense training. Their levels were significantly different between the three exercise types, reflecting the different nature and intensity of each exercise. In dogs, KC-like is a chemokine similar to human and murine CXC motif ligand 1 (CXCL1) and attracts primarily neutrophils to sites of injury or infection. CXCL1 has previously been shown to be increased in serum of mice after exercise, a response that is regulated by muscle-derived IL-6 (36). Serum IL-6 was significantly increased after ATV exercise in our study, however, it has been shown that several factors, including exercise intensity, exercise duration, and the type of muscle contraction can have an impact on the degree and timing of systemic IL-6, which in turn may be impacting serum KC-like levels in these dogs (37–41). MCP-1 is a chemokine that helps regulate migration and infiltration of monocytes and macrophages. In another study with sled dogs, MCP-1 was increased at the mid-point and immediately after the race compared to the starting concentration (42). We found that levels of MCP-1 were associated with the FPCA scores: FPCA2 was positively associated with MCP-1 and FPCA1 was positively associated with levels of MCP-1 and KC-like but only through its interaction term with the exercise type. While these associations point in the direction of an effect of the intensity of the physical stress on the temporal patterns of change of the gut microbiome, further investigation will be needed in order to better understand the nature of this interaction. In particular, the three exercises were performed at different times of the year, so that we cannot exclude seasonality or other environmental factors having contributed to the variability on the performance and the microbial community.

Our approach can be applied to describe and analyze data from any longitudinal microbiome study, providing a framework to model the evolution over time of the microbial composition in a multi-variate way. The method is therefore a valuable tool to compare and cluster subjects, based on how their microbiome responds to external perturbations. The method is flexible and can be applied, in theory, to any longitudinal study design. However, the number and the regularity of the time points will have an impact on the ability to recover the actual trajectories from the sampled data. Strategies to define optimal designs in this context have been investigated by several authors and are an area of active research (43, 44). To our knowledge, this is the first study proposing a principled approach to quantify microbiome resilience in healthy dogs. The mathematical approach is actually applicable to a different choice of host and can also be applied in human studies.

## Data Availability

The data presented in the study are deposited in the National Center for Biotechnology Information Sequence Read Archive (NCBI SRA) at https://www.ncbi.nlm.nih.gov/sra/; project accession number PRJNA1241257.
