# Supplementary material for: A bi-variate framework to model microbiome resilience in healthy dogs
Source: Front Vet Sci. 2025 Apr 2;12:1486679. doi: 10.3389/fvets.2025.1486679 (PMC12001528; doi:10.3389/fvets.2025.1486679)
Supplement: Supplementary file 1 [file Data_Sheet_1.docx]

Supplementary Material

# Supplementary Data

Supplementary Material should be uploaded separately on submission. Please include any supplementary data, figures and/or tables.

Supplementary material is not typeset so please ensure that all information is clearly presented, the appropriate caption is included in the file and not in the manuscript, and that the style conforms to the rest of the article.

# Supplementary Figures and Tables

For more information on Supplementary Material and for details on the different file types accepted, please see [here](https://www.frontiersin.org/guidelines/author-guidelines#supplementary-material).

## Supplementary Figures

**
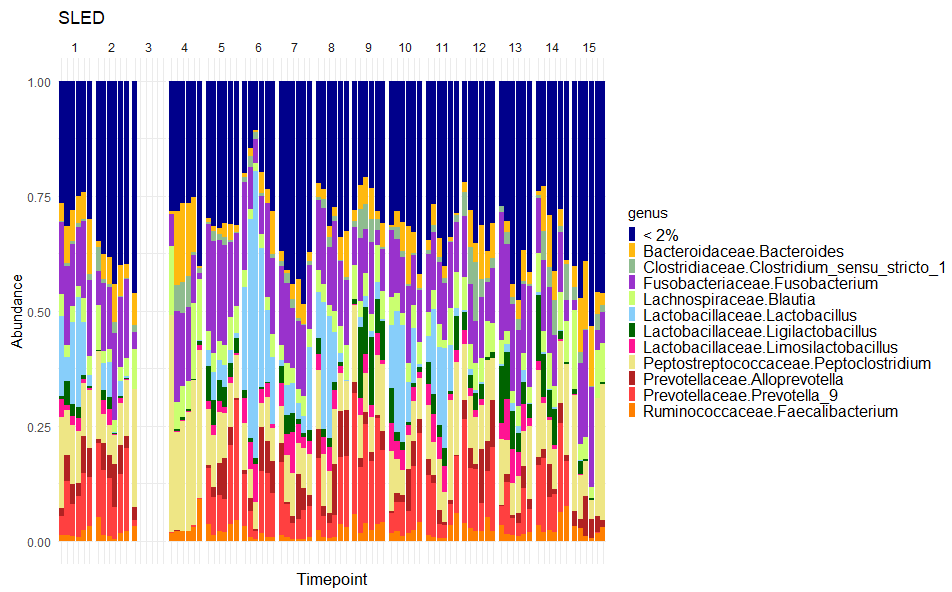
**

Supplementary Figure 1. Stacked bar plot representing the relative abundance of the microbiota, at genus level, for each dog at a time point for the sled exercise. Abundances below a threshold (0.02) were collapsed to a common category. Timepoints are ordered temporally: pre-exercise, post-exercise, 3hr post-exercise, 6hr post-exercise, 24hr washout, 48hr washout. Dog 3 missed measurements at several timepoints.


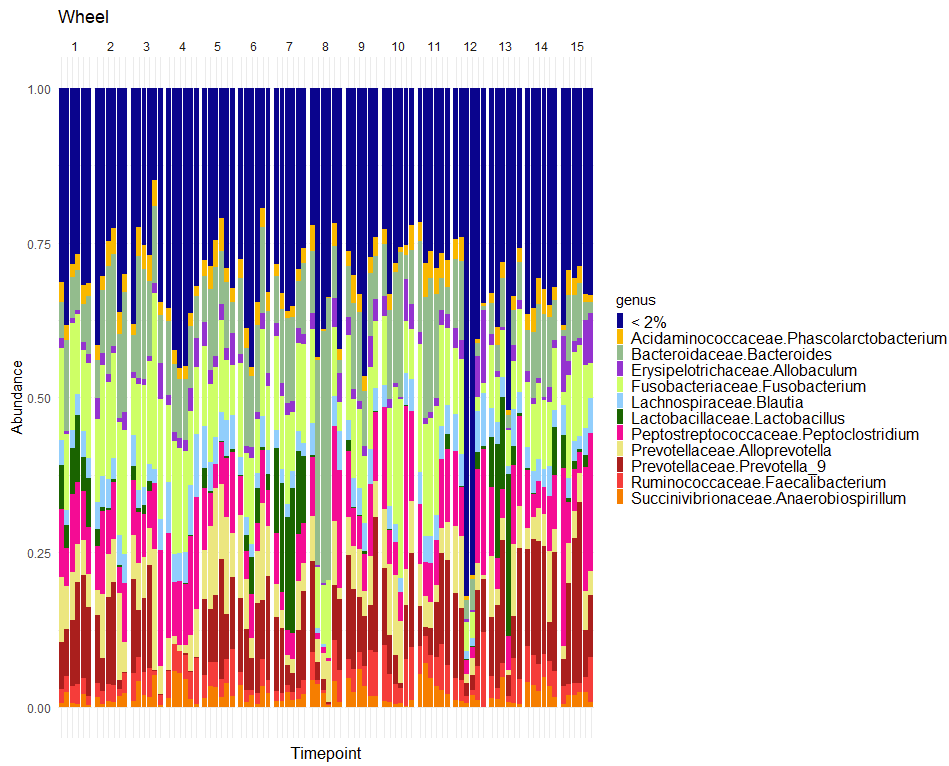


Supplementary Figure 2. Stacked bar plot representing the relative abundance of the microbiota, at genus level, for each dog at a time point for the wheel exercise. Abundances below a threshold (0.02) were collapsed to a common category. Timepoints are ordered temporally: pre-exercise, post-exercise, 3hr post-exercise, 6hr post-exercise, 24hr washout, 48hr washout.

**
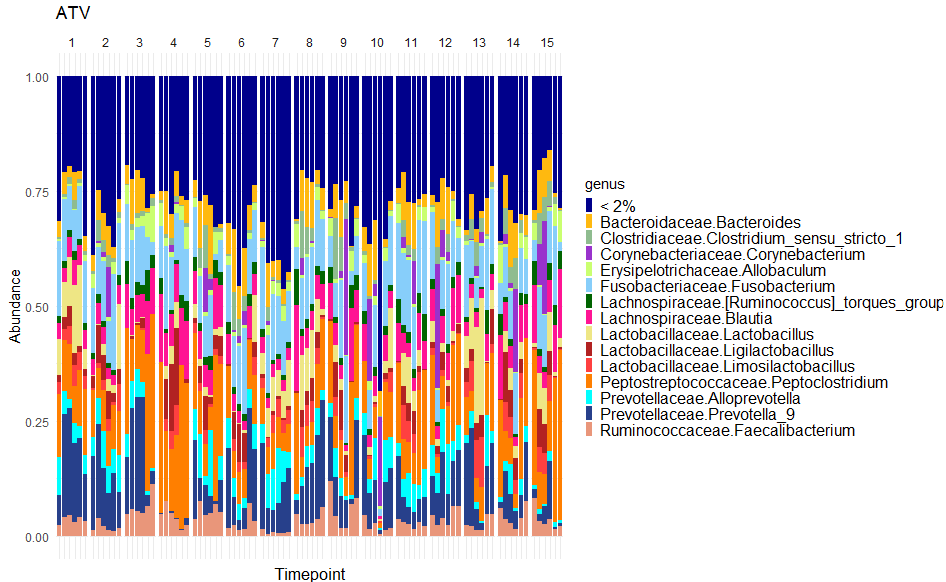
**

Supplementary Figure 3. Stacked bar plot representing the relative abundance of the microbiota, at genus level, for each dog at a time point for the ATV exercise. Abundances below a threshold (0.02) were collapsed to a common category. Timepoints are ordered temporally: pre-exercise, post-exercise, 3hr post-exercise, 6hr post-exercise, 24hr washout, 48hr washout.


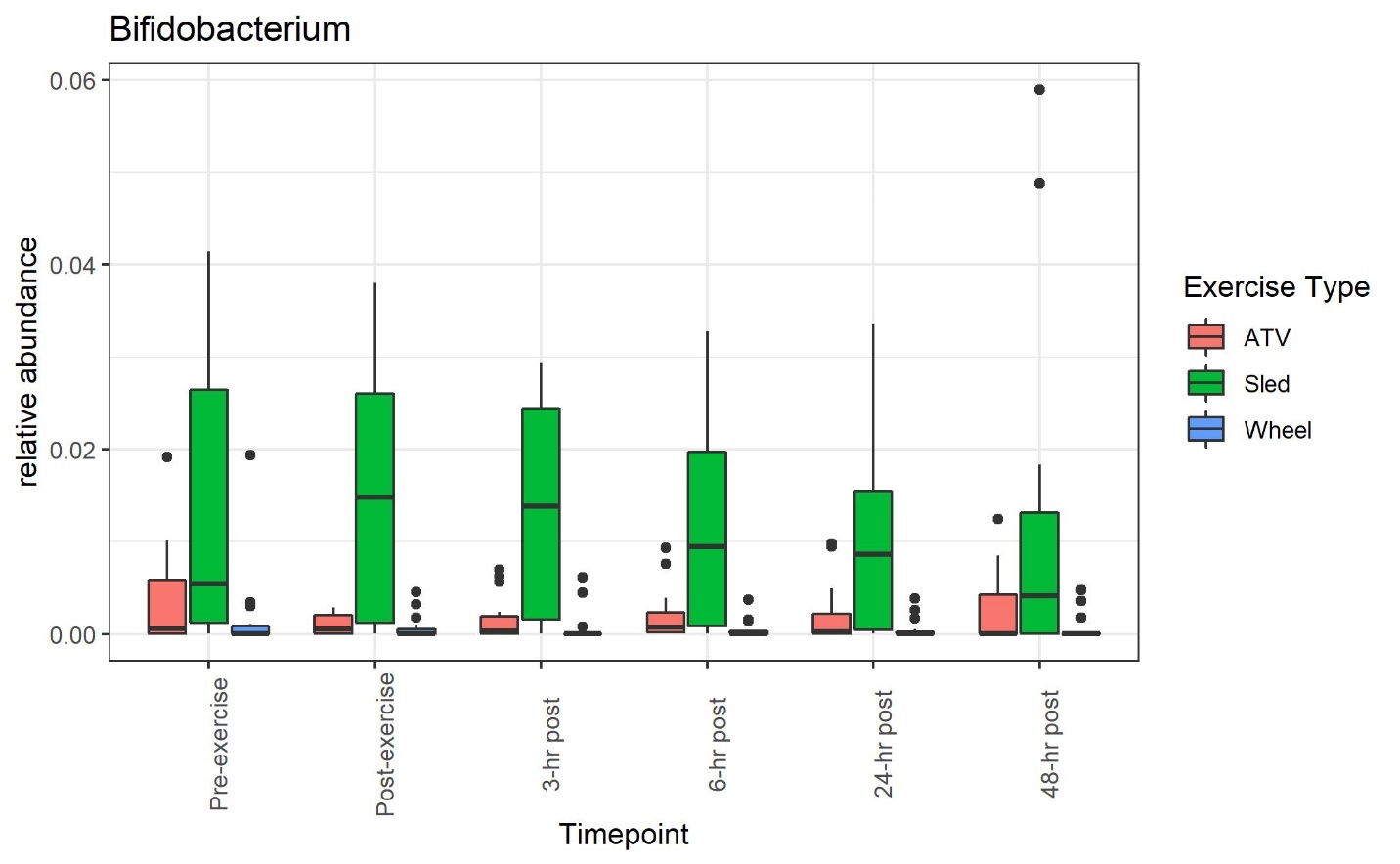
 Supplementary Figure 4. The relative abundance of the genus *Bifidobacterium* is not impacted by wheel, sled, or ATV exercise.


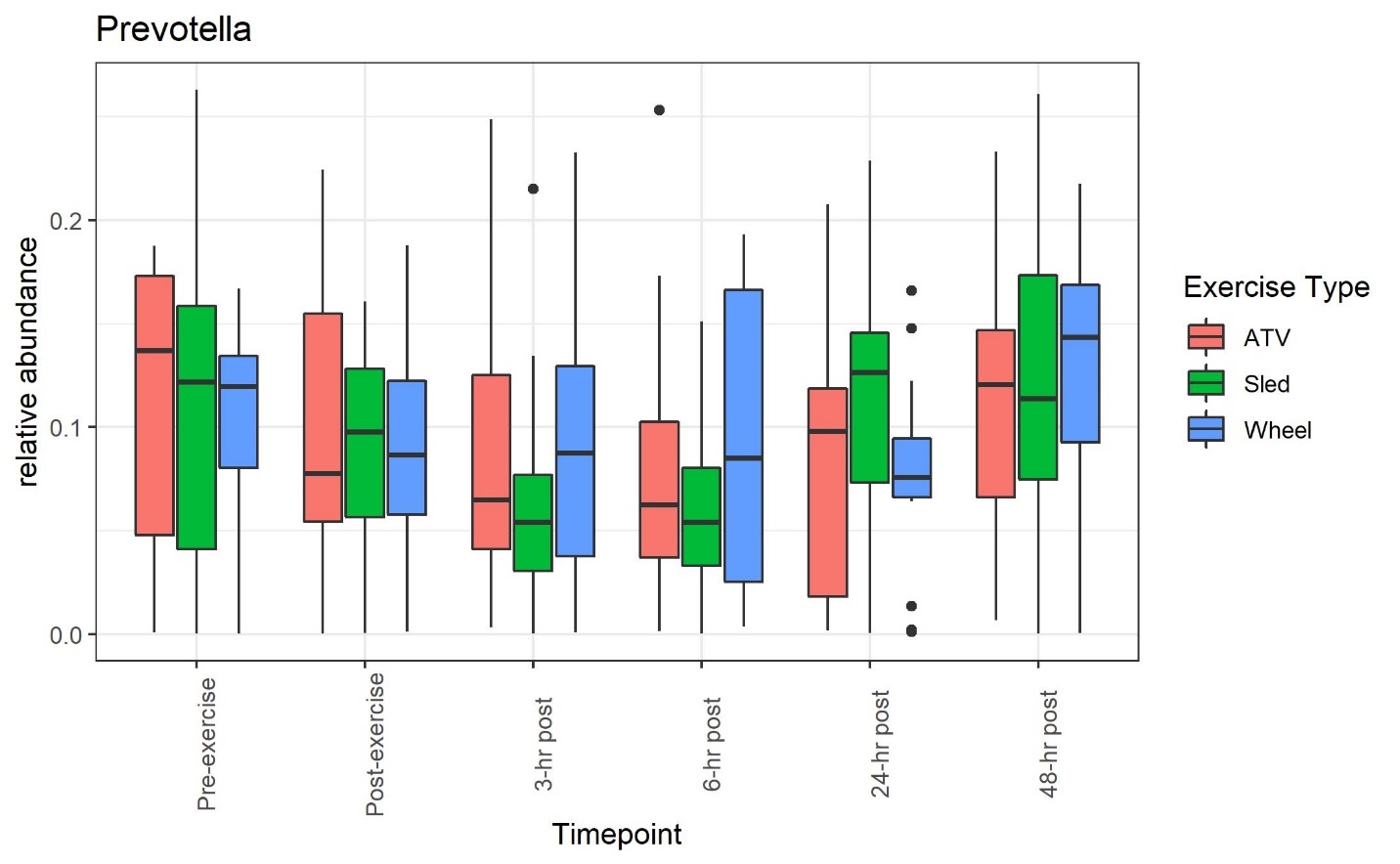


**Supplementary Figure 5.** The relative abundance of the genus *Prevotella* is not impacted by wheel, sled, or ATV exercise.

**
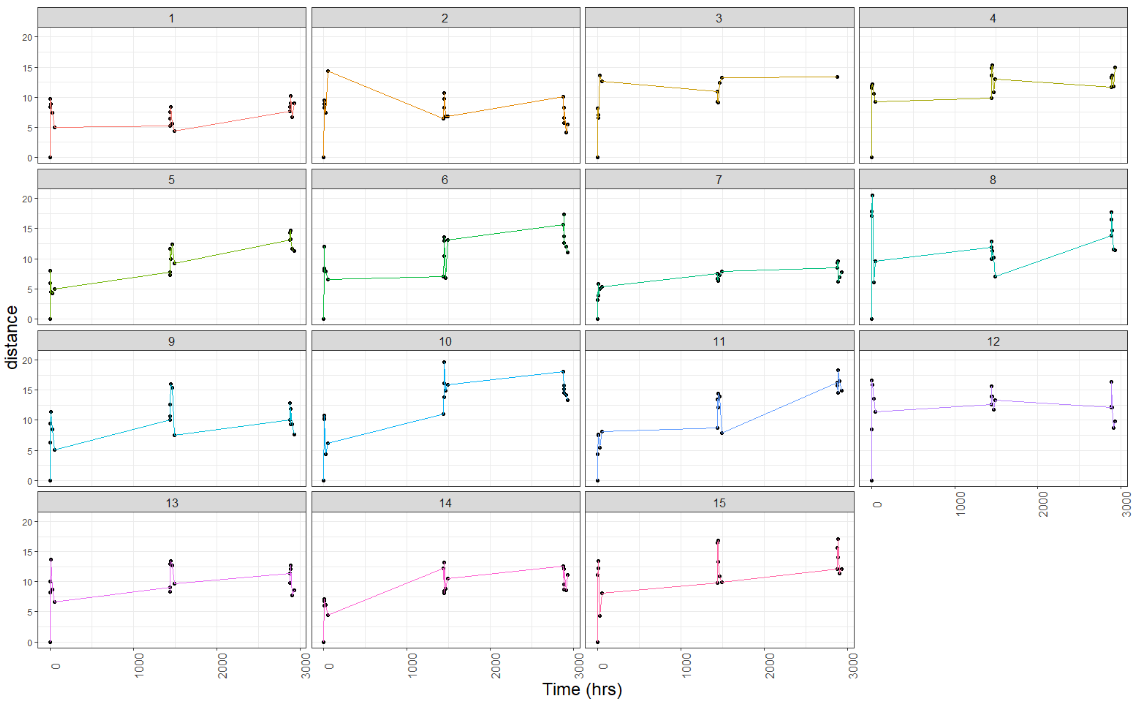
**

Supplementary Figure 6. Trajectories with distances calculated with respect to the composition before the wheel exercise. Chronologically, the wheel exercise was the first (August 2018), followed by ATV (October 2018) and Sled (February 2019). These trajectories suggest a significant change of the pre-exercise time point in the long term.

## Supplementary Tables

Supplementary Table 1

|  | **SLED** | | **WHEEL** | | **ATV** | |
| --- | --- | --- | --- | --- | --- | --- |
| **genus** | **estimate** | **FDR** | **estimate** | **FDR** | **estimate** | **FDR** |
| f__Lactobacillaceae.g__Lactobacillus | 37.43 | 0.01 | 6.34 | 0.86 | 82.94 | 0.00 |
| f__Prevotellaceae.g__Prevotella_9 | -0.29 | 0.10 | 0.22 | 0.86 | 0.58 | 0.33 |
| f__Corynebacteriaceae.g__Corynebacterium | 454.41 | 0.01 | 484.87 | 0.01 | 551.27 | 0.00 |
| f__Erysipelatoclostridiaceae.g__Catenibacterium | 0.59 | 0.76 | 3.18 | 1.00 | 2.75 | 0.89 |
| f__Peptostreptococcaceae.g__Peptoclostridium | -0.37 | 0.00 | -0.44 | 0.01 | -0.49 | 0.00 |
| f__Bacteroidaceae.g__Bacteroides | 5.58 | 0.00 | 1.25 | 0.01 | 4.79 | 0.00 |
| f__Lactobacillaceae.g__Limosilactobacillus | 19.81 | 0.29 | 25.08 | 0.33 | 49.56 | 0.00 |
| f__Prevotellaceae.g__Alloprevotella | 1.62 | 0.13 | -0.02 | 0.86 | 0.94 | 0.09 |
| f__Lactobacillaceae.g__Ligilactobacillus | 8.19 | 1.00 | 29.01 | 0.12 | 130.81 | 0.00 |
| f__Peptostreptococcaceae.g__Peptostreptococcus | 0.72 | 0.89 | 26.31 | 0.15 | 3.28 | 0.09 |
| f__Prevotellaceae.g__Prevotellaceae_Ga6A1_group | 34.50 | 0.00 | 19.43 | 0.13 | 17.49 | 0.00 |
| f__Muribaculaceae.g__Muribaculaceae | 2.63 | 1.00 | -0.48 | 0.01 | 0.29 | 0.54 |
| f__Clostridiaceae.g__Clostridium_sensu_stricto_1 | 14.01 | 0.03 | 8.39 | 0.01 | 10.39 | 0.00 |
| f__Fusobacteriaceae.g__Fusobacterium | 0.70 | 1.00 | 0.44 | 0.58 | 0.26 | 0.81 |
| f__Sutterellaceae.g__Parasutterella | 22.77 | 0.01 | 10.08 | 0.56 | 28.26 | 0.04 |
| f__Succinivibrionaceae.g__Anaerobiospirillum | 11.68 | 0.01 | 0.59 | 0.45 | 13.35 | 0.00 |
| f__Bifidobacteriaceae.g__Bifidobacterium | 0.16 | 1.00 | 1.07 | 1.00 | 0.74 | 0.11 |
| f__Lachnospiraceae.g__Blautia | -0.36 | 0.00 | -0.29 | 0.02 | -0.39 | 0.00 |
| f__Erysipelotrichaceae.g__Turicibacter | 3.78 | 0.25 | 1.65 | 0.51 | 0.13 | 0.89 |
| f__Erysipelotrichaceae.g__Allobaculum | 0.22 | 0.14 | -0.47 | 0.01 | -0.45 | 0.03 |
| f__Anaerovoracaceae.g__[Eubacterium]_brachy_group | -0.08 | 0.32 | -0.55 | 0.01 | -0.50 | 0.00 |
| f__Enterobacteriaceae.g__Escherichia-Shigella | 1.16 | 0.31 | 47.77 | 0.09 | 14.13 | 0.00 |
| f__Ruminococcaceae.g__Faecalibacterium | -0.49 | 0.00 | -0.26 | 0.06 | -0.18 | 0.19 |
| f__Burkholderiaceae.g__Ralstonia | 78.36 | 0.00 | 591.68 | 0.01 | 219.59 | 0.00 |
| f__Erysipelotrichaceae.g__uncultured | 0.22 | 0.74 | -0.15 | 0.38 | -0.35 | 0.05 |
| f__Streptococcaceae.g__Streptococcus | 0.44 | 0.80 | 71.98 | 0.09 | 6.84 | 0.00 |
| f__Sutterellaceae.g__Sutterella | 17.90 | 0.01 | 1.40 | 0.15 | 18.22 | 0.00 |
| f__Peptococcaceae.g__Peptococcus | -0.11 | 0.53 | -0.47 | 0.01 | -0.21 | 0.06 |
| f__Acidaminococcaceae.g__Phascolarctobacterium | 9.88 | 0.17 | 0.20 | 0.53 | 8.35 | 0.00 |
| f__Erysipelotrichaceae.g__Dubosiella | 1.26 | 0.11 | -0.04 | 0.39 | -0.27 | 0.11 |
| f__Erysipelatoclostridiaceae.g__Erysipelatoclostridium | -0.56 | 0.00 | -0.21 | 0.33 | 0.27 | 0.54 |
| f__Lachnospiraceae.g__[Ruminococcus]_torques_group | -0.40 | 0.03 | -0.30 | 0.06 | -0.45 | 0.00 |
| f__Lachnospiraceae.g__unknown | -0.43 | 0.02 | -0.26 | 0.19 | -0.42 | 0.00 |

Supplementary Table 1. Comparison of individual taxa before and 3 hours after exercise. FDR = false discovery rate, calculated as a Benjamini-Hochsberg correction after a Wilcoxon rank-sum test. The estimate refers to the rate of change value at 3hr / baseline – 1; therefore a positive value means an increase in the relative abundance, a negative value means a decrease in relative abundance.

**Supplementary Table 2**

|  | Pre-exercise  (N=44) | Post-exercise  (N=44) | 3 hr post-exercise  (N=44) | 6 hr post-exercise  (N=44) | 24 hr post-exercise  (N=44) | 48 hr post-exercise  (N=44) | Overall  (N=264) |
| --- | --- | --- | --- | --- | --- | --- | --- |
| **IL15** |  |  |  |  |  |  |  |
| Mean (SD) | 257 (474) | 257 (490) | 240 (421) | 263 (492) | 228 (410) | 228 (413) | 246 (446) |
| Median [Min, Max] | 38.8 [9.03, 1770] | 37.2 [0.396, 1710] | 34.5 [5.63, 1420] | 37.9 [6.01, 1680] | 35.1 [2.50, 1470] | 34.8 [2.40, 1460] | 35.6 [0.396, 1770] |
| Missing | 14 (31.8%) | 12 (27.3%) | 15 (34.1%) | 14 (31.8%) | 15 (34.1%) | 15 (34.1%) | 85 (32.2%) |
| **IL18** |  |  |  |  |  |  |  |
| Mean (SD) | 157 (312) | 162 (332) | 145 (291) | 156 (324) | 138 (271) | 130 (236) | 148 (294) |
| Median [Min, Max] | 35.3 [5.48, 1350] | 34.4 [2.62, 1360] | 30.7 [7.56, 1150] | 34.5 [6.17, 1300] | 32.5 [6.63, 1160] | 32.6 [5.97, 884] | 33.5 [2.62, 1360] |
| **IL2** |  |  |  |  |  |  |  |
| Mean (SD) | 172 (332) | 183 (365) | 163 (326) | 179 (369) | 153 (298) | 144 (245) | 166 (322) |
| Median [Min, Max] | 34.8 [2.22, 1450] | 34.5 [0.707, 1460] | 34.5 [0.418, 1290] | 34.7 [0.0735, 1460] | 32.9 [2.75, 1240] | 36.7 [1.48, 861] | 34.5 [0.0735, 1460] |
| Missing | 14 (31.8%) | 14 (31.8%) | 14 (31.8%) | 14 (31.8%) | 15 (34.1%) | 16 (36.4%) | 87 (33.0%) |
| **IL6** |  |  |  |  |  |  |  |
| Mean (SD) | 98.0 (203) | 106 (234) | 92.1 (213) | 104 (251) | 87.8 (185) | 86.3 (167) | 95.8 (209) |
| Median [Min, Max] | 19.8 [0.198, 837] | 19.2 [1.85, 1060] | 18.9 [1.37, 1120] | 20.0 [1.12, 1350] | 21.1 [0.628, 732] | 19.8 [1.43, 658] | 19.2 [0.198, 1350] |
| Missing | 2 (4.5%) | 1 (2.3%) | 1 (2.3%) | 2 (4.5%) | 3 (6.8%) | 4 (9.1%) | 13 (4.9%) |
| **IL7** |  |  |  |  |  |  |  |
| Mean (SD) | 244 (500) | 266 (543) | 232 (477) | 256 (553) | 215 (432) | 218 (407) | 239 (484) |
| Median [Min, Max] | 44.5 [0.821, 2370] | 48.5 [1.09, 2260] | 37.6 [0.243, 1970] | 34.8 [1.40, 2410] | 32.7 [0.757, 1880] | 47.1 [1.42, 1440] | 41.1 [0.243, 2410] |
| Missing | 2 (4.5%) | 3 (6.8%) | 3 (6.8%) | 3 (6.8%) | 3 (6.8%) | 4 (9.1%) | 18 (6.8%) |
| **IL8** |  |  |  |  |  |  |  |
| Mean (SD) | 4080 (1870) | 4710 (2280) | 3630 (1820) | 3300 (1680) | 3960 (1790) | 3800 (1800) | 3910 (1910) |
| Median [Min, Max] | 3650 [774, 8430] | 4340 [1100, 11100] | 3370 [724, 8670] | 2940 [642, 8190] | 3650 [874, 8480] | 3550 [973, 8500] | 3560 [642, 11100] |
| **IL10** |  |  |  |  |  |  |  |
| Mean (SD) | 152 (467) | 138 (445) | 145 (409) | 143 (424) | 142 (446) | 194 (578) | 150 (451) |
| Median [Min, Max] | 19.6 [1.10, 2440] | 32.4 [2.31, 2580] | 35.0 [8.06, 2360] | 27.9 [1.15, 2300] | 16.4 [1.70, 2340] | 26.6 [3.99, 2720] | 28.0 [1.10, 2720] |
| Missing | 16 (36.4%) | 10 (22.7%) | 10 (22.7%) | 14 (31.8%) | 16 (36.4%) | 22 (50.0%) | 88 (33.3%) |
| **IP10** |  |  |  |  |  |  |  |
| Mean (SD) | 15.0 (26.1) | 14.6 (26.1) | 14.5 (26.4) | 14.1 (25.2) | 14.5 (25.8) | 14.9 (26.3) | 14.6 (25.7) |
| Median [Min, Max] | 7.48 [0.215, 147] | 7.04 [0.279, 153] | 7.22 [0.142, 154] | 6.98 [0.218, 147] | 7.87 [0.0876, 150] | 7.75 [0.142, 152] | 7.75 [0.0876, 154] |
| Missing | 0 (0%) | 0 (0%) | 0 (0%) | 0 (0%) | 0 (0%) | 1 (2.3%) | 1 (0.4%) |
| **KC_LIKE** |  |  |  |  |  |  |  |
| Mean (SD) | 245 (130) | 297 (142) | 344 (135) | 318 (128) | 243 (124) | 254 (132) | 283 (136) |
| Median [Min, Max] | 230 [86.4, 724] | 258 [115, 777] | 309 [121, 760] | 296 [112, 620] | 237 [85.9, 616] | 232 [83.5, 752] | 257 [83.5, 777] |
| **MCP1** |  |  |  |  |  |  |  |
| Mean (SD) | 264 (104) | 328 (107) | 362 (131) | 281 (110) | 238 (94.3) | 238 (93.7) | 285 (116) |
| Median [Min, Max] | 223 [146, 602] | 296 [178, 656] | 320 [154, 691] | 248 [136, 619] | 207 [131, 584] | 214 [121, 513] | 256 [121, 691] |
| **TNF-a** |  |  |  |  |  |  |  |
| Mean (SD) | 126 (167) | 139 (180) | 132 (170) | 139 (197) | 122 (150) | 118 (135) | 129 (165) |
| Median [Min, Max] | 37.9 [1.02, 573] | 44.5 [1.32, 555] | 39.9 [4.32, 532] | 36.1 [0.712, 632] | 33.5 [3.42, 471] | 30.7 [6.27, 384] | 36.7 [0.712, 632] |
| Missing | 24 (54.5%) | 25 (56.8%) | 26 (59.1%) | 25 (56.8%) | 26 (59.1%) | 26 (59.1%) | 152 (57.6%) |

**Supplementary Table 2:** Summary of values for measured inflammatory markers. Missing values indicate measured inflammatory marker was below the limit of detection for the assay.
